# Functionalization of lipid nanoemulsions with humanized antibodies using plug-and-play cholesterol anchor for targeting cancer cells

**DOI:** 10.1039/d5na00606f

**Published:** 2025-09-23

**Authors:** Valeria Jose Boide-Trujillo, Vincent Mittelheisser, Fei Liu, Olivier Lefebvre, Bohdan Andreiuk, Nicolas Anton, Jacky G. Goetz, Andrey S. Klymchenko

**Affiliations:** a Laboratoire de Bioimagerie et Pathologies, CNRS UMR_7021, ITI SysChem-Chimie des Systèmes Complexes, Université de Strasbourg Illkirch France andrey.klymchenko@unistra.fr; b Tumor Biomechanics Lab, INSERM UMR_S1109, Fédération de Médecine Translationnelle de Strasbourg, Université de Strasbourg, Equipe labélisée Ligue Nationale Contre le Cancer Strasbourg France jacky.goetz@inserm.fr https://www.goetzlab.fr; c Regenerative Nanomedicine (RNM), INSERM UMR_1260, Fédération de Médecine Translationnelle de Strasbourg, Université de Strasbourg Strasbourg France

## Abstract

Lipid nanoemulsions (NEs) are promising green nanocarriers for diagnostic and therapeutic applications, but their functionalization with biomolecules, such as antibodies, remains a challenge due to liquid nature of their core. Here, we developed an original plug-and-play strategy to graft an antibody (trastuzumab) at the surface of NEs, using components generally recognized as safe (GRAS). We synthesized a reactive 4-nitrophenyl carbonate of cholesterol (NPC-Chol) and a Biotin-PEG_3000_-Lysine linker, which can react within one-pot formulation to form an amphiphilic carbamate Biotin-PEG_3000_-Cholesterol. The cholesterol ensures anchorage of the linker, which effectively exposes biotin moiety at the surface of NEs for further antibody grafting using a biotin–neutravidin coupling. The reaction between the Biotin-PEG_3000_-Lysine linker and NPC-Chol was confirmed by ^1^H-NMR and absorption spectroscopy. The obtained biotinylated 50-nm NEs loaded with a near-infrared dye were successfully targeted to neutravidin-coated glass surfaces and imaged at the single-droplet level. The biotinylated NEs bearing the trastuzumab antibody targeted specifically HER2-amplified breast cancer models HCC-1954 and SKBR3, in contrast to control MDA-MB-231 (HER2-low) cells. Altogether, our study proposes an efficient methodology for grafting antibodies to the surface of NEs, which offers new opportunities of application of these green nanocarriers in biomedicine.

## Abstract 2

A plug-and-play functionalization of nanoemulsions with antibodies based on reactive cholesterol and bifunctional linker was proposed for specific cell targeting.

## Graphical abstract

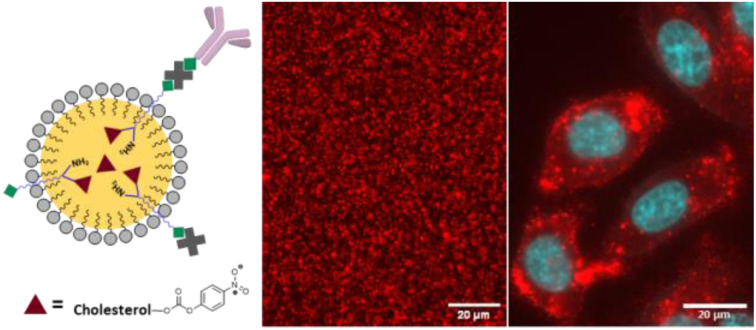

## Introduction

Cancer nanomedicine has initially emerged to improve the delivery selectivity of chemotherapeutics to reduce associated toxicities.^1,2^ Among existing nanoparticles (NPs), nanoemulsions (NEs) constitute sustainable, biomimetic nanomaterials mainly composed of lipids and PEGs, which are materials generally recognized as safe (GRAS).^3–5^ Their oil core serves as an ideal reservoir for encapsulating lipophilic compounds, including cytotoxic drugs and imaging agents.^6–8^ In particular, encapsulation of fluorescent dyes transforms them into bright fluorescence imaging agents.^6^ Dye-loaded NEs enable imaging vasculature and tumors,^8^ crossing the brain blood barrier^9^ and intranasal brain delivery^10^ as well as tracking individual particles^11^*in vivo*. Their long circulation time and potential of therapeutic molecules loading into their core render them particularly attractive for *in vivo* imaging and drug delivery.^6–8^ These nanocarriers are known to passively accumulate in the tumor through the enhanced permeability and retention (EPR) effect.^8,12^ However, as defined in meta-analyses, this passive targeting remains suboptimal and thus requires implementing strategies for specific targeting.^13,14^

Enhancing the targeting selectivity of drugs and contrast agents is a primary focus in cancer diagnostics and treatment to minimize systemic exposure and increase their efficacy.^15^ One major strategy has been the development of antibody-drug conjugates (ADCs), which merge the targeting specificity of monoclonal antibodies with the potency of cytotoxic drugs. These features have led to the widespread clinical adoption of ADCs, with routine applications for the treatment of hematological malignancies and HER2-amplified breast cancers due to their strong antitumoral activity.^16^ However, to maintain favorable pharmacokinetic and pharmacodynamic profiles, ADCs have relatively low drug/antibody ratios (≈4 molecules/antibody).^17^ To overcome this limitation, nanoparticles (NPs) delivery systems have been explored, which can carry >10 times as many small molecules as ADCs.^2,18^ The same concerns contrast agents, where single NP can carry molecules, such as fluorescent dyes, this enhancing sensitivity of detection.^19,20^ As such, combining the targeting selectivity of antibodies with the drug/contrast agent loading ability of NPs seems a logical step to advance this field. We recently confirmed the interest in developing such targeted formulations by highlighting that antibody-targeted nanoparticles demonstrate greater tumor uptake than non-decorated ones.^14^ However, functionalization of NEs with antibodies has been poorly explored to date.^6^

The major challenge in surface modifications of NEs is the dynamic nature of their liquid/liquid interface of NEs, which renders surface grafting poorly stable. In previous studies, NEs were functionalized with a peptide arginylglycylaspartic acid (RGD) using lipids^21^ or fatty acids,^22^ allowing the increased targeting of α_v_β_3_ integrin-rich cells. However, this approach does not guarantee stable grafting for antibodies, because lipid/fatty acid residues may exchange with other lipid components of serum, as for instance was shown for other lipid nanoparticles.^23^ To overcome this limitation, we recently proposed to use NEs with amphiphilic polymers bearing biotin^24^ or an antibody.^25^ However, the limitation here is the use of non-biodegradable polymer, which raises concerns about the long-term toxicity of this approach. Overall, antibodies are an excellent targeting ligand for delivery and imaging applications because of high affinity and selectivity to the target.^26,27^ They were found highly efficient for targeting different NPs in drug delivery applications.^14,28,29^ To achieve effective functionalization of NEs with antibodies and preserve GRAS characteristics and biomimetic nature, a “green” approach should be developed, which would involve exclusively natural/biocompatible components.

In the present work, we engineered antibody-targeted NEs to enhance tumor cell selectivity and improve their potential for clinical translation. To this end, we used a biotinylation strategy, which is commonly used in molecular biology and bio-nanotechnology for its rapid, efficient, and robust bio-conjugation.^30,31^ We synthesized a reactive derivative of cholesterol and a Biotin-PEG_3000_-Lysine linker, which can react within a one-pot formulation to form an amphiphilic carbamate Biotin-PEG_3000_-Cholesterol. The cholesterol moiety was expected to ensure its firm anchorage in NEs, whereas the long hydrophilic PEG_3000_ chain was expected to expose the biotin moiety at the surface of NEs for further antibody grafting using a biotin–neutravidin coupling. Successful NE biotinylation was confirmed by fluorescence staining on a glass surface. These biotinylated-NEs were further functionalized with biotinylated antibodies using neutravidin as a bridge. We selected trastuzumab as the targeting antibody due to its routine use as part of the therapeutic arsenal as a single agent or in ADCs formulations to treat HER2-amplified patients.^32^ Trastuzumab-functionalized NEs effectively recognized their cognate receptors and selectively accumulated into HER2-expressing cancer cell lines (in comparison to HER2-low ones). Overall, this study presents a highly biocompatible, modular, and stable way to functionalize NEs surfaces with antibodies. These antibody-targeted NEs demonstrated enhanced and antigen-selective tumor cell targeting *in vitro*, suggesting that NEs are promising vehicles for targeted delivery and imaging.

## Materials and methods

### Materials

Boc-NH-PEG-NH_2_ (3 kDa) was purchased from Iris Biotech GmbH. Biotin (97%) and diBoc-lysine (98%) were acquired from abcr GmbH. 2-(7-Aza-1*H*-benzotriazole-1-yl)-1,1,3,3-tetramethyluronium hexafluorophosphate (HATU) was provided by Fluorochem. *N*,*N*-Diisopropylethylamine (DiPEA) (>99%), Kolliphor^®^ ELP, triethylamine, butylamine (99.5%), and BSA-biotin were supplied by Sigma-Aldrich. Trifluoro acetic acid (TFA) (99%), NHS-biotin, and neutravidin were obtained from ThermoFisher. Trastuzumab (Herceptin®) was purchased from the ICANS Pharmacy. Vitamin E acetate (>97.0%) was acquired from TCI. Dulbecco's phosphate-buffered saline (DPBS) (without Ca^2+^ and Mg^2+^) was provided by Dominique Dutscher. Buffer preparations and formulation of lipid nanoemulsions (NEs) were done with filter-sterilized Milli-Q water. Borate buffer (pH 8.4) was prepared with sodium tetraborate (25 mM), NaCl (25 mM), and EDTA (1 mM). Phosphate buffer (pH 8.5) was prepared with dipotassium hydrogen phosphate (40 mM) and NaCl (150 mM). Final pH values were adjusted with 1 M hydrochloric acid or 1 M sodium hydroxide. For zeta potential measurements, NEs were diluted into 10 mM NaCl solution prepared with MilliQ water.

### Equipment

Size distributions and zeta potential of NEs were determined by dynamic light scattering using a Zetasizer Nano ZSP (Malvern Instruments). Absorption spectra were recorded on a Cary 5000-UV-vis-NIR spectrophotometer (Agilent). NMR spectra were recorded at 20 °C on a Bruker Avance III 400 MHz NMR spectrometer (Bruker). Conjugation experiments were carried out in DNA low-binding tubes 1.5 mL (Eppendorf) at atmospheric pressure at room temperature unless otherwise stated. Ultrafiltration was carried out in centrifugal filters Amicon^®^ Ultra 0.5 mL (Merck Millipore) with a molecular weight cut-off (MWCO) of 50 kDa. Centrifugation was carried out on a MiniSpin plus (Eppendorf) operating at 10 000 rpm at room temperature. Size exclusion chromatography was performed on an ÄKTA start (Cytiva) operating at a flow speed of 0.5 mL min^−1^. Sephacryl S-300 High-Resolution (GE Healthcare) resin was used to pack the column. Epifluorescence microscopy images were recorded on an ECLIPSE Ti (Nikon). Confocal microscopy was performed on an inverted Olympus Spinning-disk.

### Synthesis

#### Compound 1

Biotin (5 eq., 62.3 mg, 0.25 mmol) and HATU (5 eq., 100.0 mg, 0.25 mmol) were dissolved in 5 mL of dry DMF. DiPEA (18 eq., 145 μL, 0.85 mmol) was added and stirred for 15 min. A 5 mL solution of Boc-NH-PEG-NH_2_ (1 eq., 140.0 mg, 0.03 mmol) in dry DMF was added to the previous mixture, and the final mixture was stirred overnight at 40 °C. The crude was purified by size exclusion chromatography LH-20 (DCM : MeOH, 1 : 1) to afford compound 1 (107.0 mg, 0.03 mmol, 73%) as a colorless wax. ^1^H-NMR (400 MHz, CD_3_OD) *δ* 4.50 (m, 1H), 4.31 (dd, *J* = 4.4, 7.8 Hz, 1H), 3.81 (t, *J* = 4.8 Hz, 2H), 3.70–3.58 (m, ∼285H), 3.55 (m, 2H), 3.51 (t, *J* = 5.7 Hz, 1H), 3.46 (t, *J* = 4.7 Hz, 1H), 3.36 (m, 2H), 3.22 (p, *J* = 5.7 Hz, 3H), 2.93 (dd, *J* = 5.0, 12.7 Hz, 1H), 2.71 (d, *J* = 12.7 Hz, 1H), 2.23 (t, *J* = 7.4 Hz, 2H), 1.81–1.54 (bs, 4H), 1.48–1.41 (10H).

#### Compound 2

Compound 1 (1 eq., 107.0 mg, 0.03 mmol) was dissolved in dry DCM (2 mL). Trifluoroacetic acid (150 eq., 0.34 mL, 4.48 mmol) was added, followed by 3 hours of stirring at room temperature. DCM was removed under reduced pressure. Co-evaporation with methanol (1 mL) was done several times to remove the remaining trifluoroacetic acid, affording compound 2 (102.0 mg, 0.03 mmol, 96%) as light-yellow wax. ^1^H-NMR (400 MHz, CD_3_OD) *δ* 4.50 (dd, *J* = 4.9, 7.9 Hz, 1H), 4.31 (dd, *J* = 4.5, 7.9 Hz, 1H), 3.74–3.35 (m, ∼278H),3.83–3.75 (m, 4H), 3.54 (t, *J* = 5.5 Hz, 2H), 3.46 (m, 1H), 3.36 (m, 3H), 3.20 (m, 3H), 2.93 (dd, *J* = 5.0, 12.8 Hz, 1H), 2.71 (d, *J* = 12.7 Hz, 1H), 2.23 (t, *J* = 7.3 Hz, 2H), 1.77–1.53 (bs, 4H), 1.46 (q, *J* = 7.5 Hz, 2H).

#### Compound 3

DiBoc-lysine (3 eq., 51.4 mg, 0.09 mmol) and HATU (2.5 eq., 31.0 mg, 0.07 mmol) were dissolved in 5 mL of dry DMF. DiPEA (10 eq., 55 μL, 0.32 mmol) was added and stirred for 15 min. A 5 mL solution of compound 2 (1 eq., 102.0 mg, 0.03 mmol) in dry DMF was added to the previous mixture, and the final mixture was stirred overnight at 40 °C. The crude was purified by size exclusion chromatography LH-20 (DCM : MeOH, 1 : 1) to afford compound 3 (105.0 mg, 0.03 mmol, 95%) as a colorless wax. ^1^H-NMR (400 MHz, CD_3_OD) *δ* 4.50 (dd, *J* = 4.8, 7.7 Hz, 1H), 4.31 (dd, *J* = 4.4, 7.9 Hz, 1H), 3.99 (m, 1H), 3.81 (m, 2H) 3.70–3.58 (m, ∼315H), 3.54 (t, *J* = 5.4 Hz, 5H), 3.45 (m, 2H), 3.37 (q, *J* = 5.5 Hz, 4H), 3.21 (m, 1H), 3.03 (t, *J* = 5.9 Hz, 2H), 2.93 (dd, *J* = 5.0, 12.7 Hz, 1H), 2.72 (d, *J* = 12.7 Hz 1H), 2.23 (t, *J* = 7.4 Hz, 2H), 1.81–1.54 (m, 7H), 1.52–1.40 (m, 25H).

#### Compound 4

Compound 3 (1 eq., 105.0 mg, 0.03 mmol) was dissolved in 2 mL of dry DCM. Trifluoroacetic acid (150 eq., 0.34 mL, 4.48 mmol) was added, followed by 3 hours of stirring at room temperature. DCM was removed under reduced pressure. Co-evaporation with methanol (1 mL) was done several times to remove the remaining trifluoroacetic acid, affording compound 4 (104.0 mg, 0.03 mmol, 99%) as light-yellow wax. ^1^H-NMR (400 MHz, CD_3_OD) *δ* 4.49 (dd, *J* = 4.8, 8.0, 1H), 4.30 (dd, *J* = 4.3, 7.8, 1H), 3.95 (t, *J* = 6.6 Hz, 1H), 3.8 (t, *J* = 4.8 Hz, 2H) 3.71–3.56 (m, ∼306H), 3.54 (t, *J* = 5.4 Hz, 3H), 3.45 (m, 2H), 3.35 (m, 2H), 3.20 (dt, *J* = 5.2, 9.9 Hz, 1H), 3.02 (t, *J* = 7.9 Hz, 2H), 2.92 (dd, *J* = 5.0, 12.8 Hz, 1H), 2.70 (d, *J* = 12.4 Hz, 1H), 2.22 (t, *J* = 7.3 Hz, 2H), 1.91 (q, *J* = 7.9 Hz, 2H), 1.80–1.55 (m, 6H), 1.55–1.40 (m, 4H). Mass data: C_144_H_286_N_6_O_66_S (exact mass expected 3187.92). Mass detected: 1617.70 and 1086.09 (calc.: (M + 2Na^+^)/2 = 1616.96 and (M + 3Na^+^)/3 = 1085.64).

### General protocol for formulation of nanoemulsions (NEs)

NEs were produced by spontaneous nanoemulsification: 55 mg vitamin E acetate containing 6% of NPC-cholesterol with Cy5.5-TPB at 2% and 45 mg Kolliphor^®^ ELP are mixed at 60 °C for 5 min. To the oil : surfactant mixture, 230 μL of filtered Milli-Q water is added, followed by 30 s of shaking with vortex, and 10 min with ThermoShaker (60 °C, 1400 rpm). To determine encapsulation efficiency, 10 μL of NEs were diluted into a total volume of 750 μL of MilliQ water and dialyzed against 1 L of MilliQ water for 20 h. Absorption spectra were recorded before and after dialysis, using a 1 : 1001 dilution.

### Nanoemulsion biotinylation

55 mg vitamin E acetate containing 6% of NPC-cholesterol (with or without Cy5.5-TPB at 2%), Biotin-PEG-Lysine (5 mg), and triethylamine (1.3 μL) were mixed in THF (500 μL), overnight at 60 °C. THF and triethylamine were removed under reduced pressure. Kolliphor^®^ ELP (45 mg) and filtered Milli-Q water (460 μL) were added according to the general protocol for nanoemulsion formulation. Then, 66 μL of biotinylated-NEs were diluted into 100 μL phosphate buffer (pH 8.5). Butylamine (1.6 μL) was added, followed by 2 hours of incubation on a ThermoShaker (room temperature, 400 rpm). Absorbance spectra were recorded before and after the addition of butylamine to determine the extent of the reaction with the biotin-linker. Finally, biotin-NEs were purified by dialysis in Milli-Q water using a 14 kDa MWCO membrane.

### NPC-cholesterol conversion

NPC-cholesterol (3.3 mg), Biotin-PEG-Lysine (5 mg), and triethylamine (1.3 μL) were mixed in THF (500 μL), overnight at 60 °C. THF and triethylamine were removed under reduced pressure. The residue was dissolved in CDCl3 (100 μL) and transferred to a 3 mm diameter tube for ^1^H-NMR acquisition.

### Trastuzumab conjugation with biotin-NHS ester

500 μL of Trastuzumab (32 μM in borate buffer) was mixed with 5 μL of NHS-biotin (100 mM in DMSO). The reaction was incubated overnight on a ThermoShaker (room temperature, 300 rpm). Afterward, purification was done by ultrafiltration (50 kDa MWCO) using PBS. The biotin-trastuzumab was stored at 4 °C in DPBS, in the presence of 0.05% (w/v) of sodium azide, for conservation.

### Nanoemulsion functionalization with trastuzumab

200 μL of neutravidin (22 μM in DPBS) and 200 μL of biotinylated antibody (11 μM in DPBS) were incubated for 24 hours at room temperature in a DNA low-binding Eppendorf. Then, 100 μL of biotinylated-NEs were added, followed by 24 hours of further incubation. Finally, antibody-NEs were purified by size exclusion chromatography using Sephacryl S300 HR gel column, which is specially adapted for separating relatively large macromolecules or globular proteins in the range 10 to 150 kDa, and DPBS as an eluent.

### Fluorescence correlation spectroscopy (FCS) of NEs

FCS measurements were performed on a home-built confocal setup based on a Nikon inverted microscope with a Nikon 60× 1.2 NA water immersion objective. Excitation was provided by a continuous wave laser diode at 638 nm (Oxxius LCC) and photons were detected with a fibered avalanche photodiode (APD SPCM-AQR-14-FC, PerkinElmer) connected to an online hardware correlator (ALV7000-USB, ALV GmbH, Germany). The data were analyzed using the PyCorrFit software. NEs (non-functionalized or biotin NEs) in a total volume of 500 μL (DPBS or 10% FBS in DPBS) were incubated for 1 h at 37 °C. The resulting solutions were further diluted 10-fold in PBS. Alexa 647 acid was used as a reference (50 nM aqueous solution).

### Biotin-NEs immobilization protocol

8-well LabTek^®^ chambers were treated with 500 μL KOH (1 M) each and incubated for 30 min. Similar treatments were done with 100 μL BSA-biotin (0.5 mg mL^−1^) and 100 μL neutravidin (0.5 mg mL^−1^), each followed by 30 min incubation. Finally, the chambers were treated with biotinylated and non-biotinylated NEs as control, followed by 30 min incubation in darkness. Both biotinylated and non-biotinylated NEs were loaded with Cy5.5-TPB (2% with respect to the oil). All treatments were done at room temperature with DPBS washings in between.

### Cell experiments

#### Cell culture

MDA-MB-231 (ATCC #CRM-HTB-26) and HCC-1954 (ATCC #CRL-2338) human breast cancer cell lines were kindly provided by M. Donzeau (IGBMC, Strasbourg, France). Cells were cultured in RPMI1640 with l-glutamine, supplemented with 10% fetal calf serum and penicillin (100 U mL^−1^) and streptomycin (100 μg mL^−1^) and maintained at 37 °C in a humidified atmosphere containing 5% CO_2_. Receptor status was routinely assessed by flow cytometry: MDA-MB-231 cells represent epithelial-like triple-negative breast adenocarcinoma, while HCC-1954 are epithelial HER2-amplified ductal carcinoma cells.

#### Cytotoxicity

2500 MDA-MB-231 and 7500 HCC-1954 were plated in a 96-well plate and let adhere overnight. The day after, medium was changed for medium containing increasing concentration of bare NEs or antibody-targeted NEs. After 72 h, cells were washed, fixed with PFA 4% and stained for 1 h with 2% cristal violet. Excess of cristal violet was washed with tap water and plates were let dry before destaining by acetic acid. Cristal violet absorbance was measured at 595 nm using a MultiSkan FC plate-reader.

#### Nanoemulsion internalization

30 000 MDA-MB-231 or HCC-1954 were plated in an 8-well LabTek slide and let adhere overnight. The day after, medium was changed for medium containing 1.1 nM of bare NEs or antibody-targeted NEs in Opti-MEM. After 4 h of incubation at 37 °C, cells were washed and their membranes were stained with wheat germ agglutinin (WGA) Alexa-Fluor 488 (5 μg mL^−1^ in PBS) over 5 min at 37 °C. After an additional wash, cells were fixed in PFA 4% and mounted in Fluoromount/DAPI. Slides were imaged using a 60× water-immersion objective on an inverted OIympus Spinning-disk. The comparison of SKBR3 *vs.* MDA-MB-231 was done in another set of experiments. SKBR3 and MDA-MB-231 cells were grown in Dulbecco's modified Eagle medium (DMEM, Gibco) supplemented with FBS (10%), sodium pyruvate (1 mM), l-glutamine (4 mM), and phenol red. For imaging experiments, cells were seeded in 35 mm glass bottom dishes (Ibidi) and incubated over 2 days, at 37 °C under a humidified 5% CO_2_ atmosphere. Then, cells were washed with DPBS, followed by 30 min incubation in darkness with Hoechst 33 342. After washing with DPBS, cells were incubated with Antibody-NEs (0.2 nM in Opti-Mem) on ice for 1 hour. Finally, they were washed with OptiMem.

### Statistical analysis

Statistical analysis was performed with GraphPad Prism 9 software. The normal distribution of the data was tested using the Shapiro–Wilk normality test. When comparing two groups, a Mann–Whitney analysis were used, for more than 2 groups a Kruskal–Wallis test followed by the original FDR method of Benjamini and Hochberg *post*-test was used. *p* < 0.05, *; *p* < 0.01, **; *p* < 0.001, ***; *p* < 0.0001, ****. Data in graphs are presented as mean ± standard deviation.

## Results and discussion

### Design and synthesis of GRAS-compatible building blocks for NEs surface functionalization

**Fig. 1:**
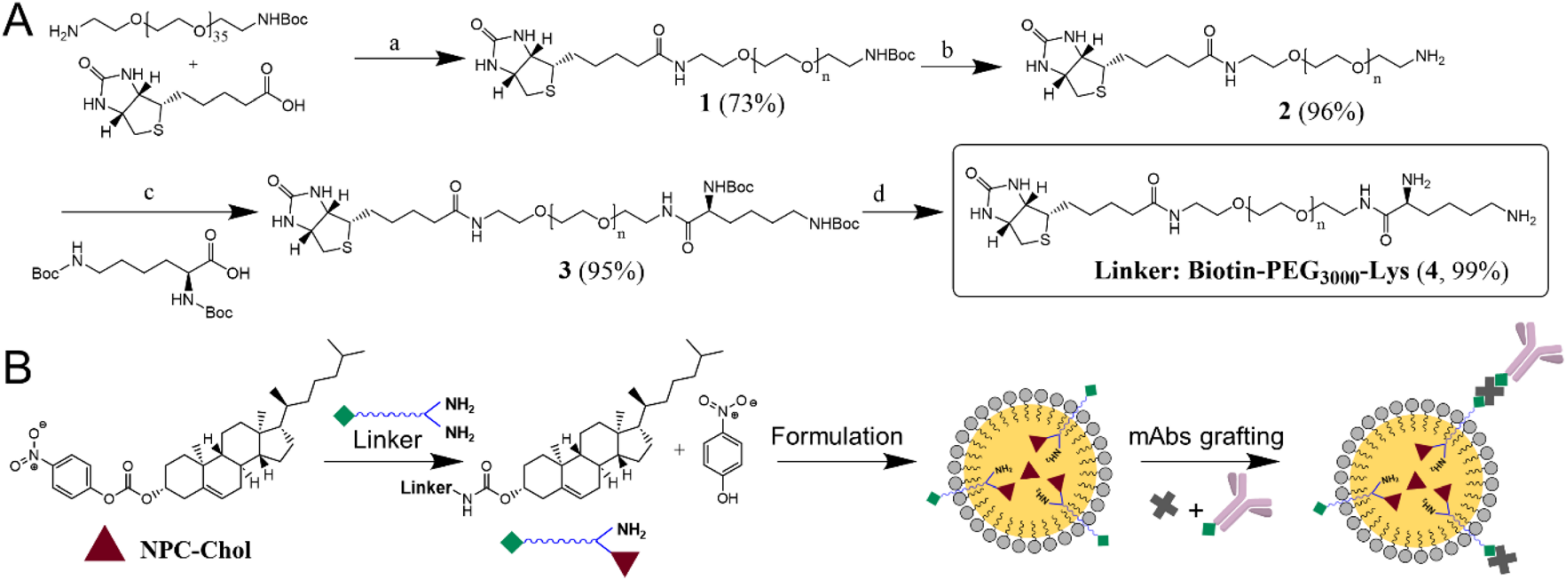
Schemes for preparation of functionalized NEs. (A) Synthesis of the Biotin-PEG_3000_-Lysine linker: (a) biotin, HATU, DiPEA, DMF, 40 °C, overnight; (b) TFA, DCM, rt, 3 h; (c) Boc-lysine(Boc)-OH, HATU, DiPEA, DMF, 40 °C, overnight; (d) TFA, DCM, rt, 3 h. (B) Scheme of plug-and-play biotinylation of lipid nanoemulsions. Reaction between the Biotin-PEG_3000_-Lysine linker and the NPC-Chol, generating the Biotin-PEG_3000_-Chol conjugate and releasing *p*-nitrophenol, followed by formation biotinylated NEs and finally grafting of neutravidin (a black cross) and biotinylated antibody.

To address the current limitations of anti-HER2 ADCs and NPs, we aimed to develop trastuzumab-functionalized NEs capable of targeting their cognate antigen expressed on the surface of tumor cells. However, given the liquid nature of the NE oil core, efficient surface anchoring of large biomolecules such as the humanized IgG_1_ antibody trastuzumab requires conjugation to a highly hydrophobic anchor. To this end, we adopted a plug-and-play approach using GRAS compounds by designing a reactive cholesterol derivative that can firmly anchor a functional linker within a one-pot protocol. Cholesterol's strong hydrophobic character and biocompatibility make it an attractive anchor for functionalization with an antibody at the NE surface. To this end, cholesterol was converted into reactive (4-nitrophenyl) carbonate of cholesterol (NPC-Chol) in one step by reaction with *p*-nitrophenyl chloroformate under basic conditions.^33^ Biotin-PEG_3000_-Lysine linker, which was synthesized in several steps (see SI) (Fig. 1). Biotin was reacted with mono-Boc-protected PEG_3000_ diamine to give compound 1, which, after Boc removal in TFA, yielded compound 2. Compound 2 was further reacted with di-Boc-protected lysine to produce conjugate 3. Then, the Boc groups of conjugate 3 were deprotected in TFA, yielding the final Biotin-PEG_3000_-Lysine linker (compound 4). The identity of each synthesis intermediate as well as the final compound was confirmed by ^1^H-NMR and mass spectrometry (Fig. S1–S5). This linker is composed of three GRAS blocks. The lysine moiety, among the most common amino acids, is expected to react with NPC-Chol through their amino groups, thus anchoring the linker in the NE oil core after formulation. The spacer made of PEG, a widely used component in drug delivery, would allow exposure of derivatizable moieties away from the NE surface. Biotin, a water-soluble vitamin, is a convenient handle for further functionalization. Then, biotinylated antibody is coupled to biotinylated-NEs through a neutravidin (avidin analogue) bridge (Fig. 1). The selected antibody was trastuzumab, which is a commonly used therapeutic antibody for targeting HER2 receptor. Altogether, we designed and synthesized a GRAS, derivatizable anchor-linker system for subsequent conjugation with targeting ligands such as trastuzumab.

### Formulation of *in situ* biotinylated lipid nanoemulsion

**Fig. 2:**
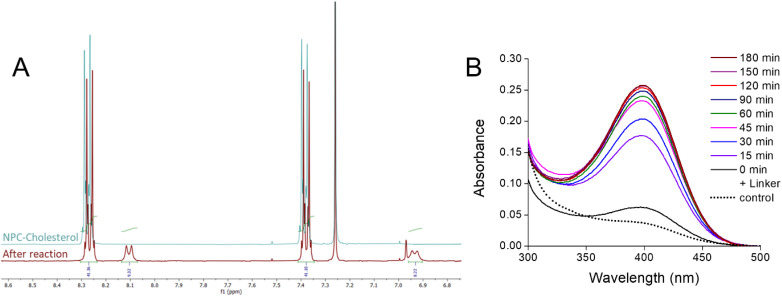
Characterization of the reaction of NPC-Chol with the biotin linker. (A) Superimposed ^1^H-NMR spectra of the NPC-Chol aromatic region, before and after the reaction with the Biotin-PEG_3000_-Lysine linker. (B) Absorption spectra of the released *p*-nitrophenol after formation of NEs with the linker (0 min + linker) after further quenching of the remaining NPC-Chol by *n*-butylamine at different time points. The control experiment was done without Biotin-PEG_3000_-Lysine.

In our approach, the reactive anchor NPC-Chol was first coupled with the linker Biotin-PEG_3000_-Lysine in THF in the presence of a base, trimethylamine, and vitamin E acetate, which will serve as the oil core later on (Fig. 1B). After evaporation of the solvent and the base, the ^1^H-NMR spectra of the reaction mixture showed new peaks, corresponding to the liberated *p*-nitrophenol (Fig. 2A). The integral of the new peaks suggested 18.5% conversion of the used NPC-Chol. Considering that complete reaction of one amino group of the linker with NPC-Chol would result in 23% conversion of the latter, the observed 18.5% conversion suggests that, under these conditions, we grafted one cholesterol anchor per linker. Then, surfactant and water were added to the oil mixture containing the *in situ*-generated Biotin-PEG_3000_-Chol, under intense stirring in order to obtain NEs by spontaneous nanoemulsification. In this previously developed method, an optimal ratio of oil (vitamin E acetate) and surfactant (Kolliphor® ELP) ensures spontaneous formation of NEs of controlled size after addition aqueous phase at a given temperature.^4,6,8^ Thus, in the control sample (non-functionalized NEs), where vitamin E acetate oil (containing 6 wt% NPC-Chol and 2 wt% of a fluorescent dye) was mixed with Kolliphor® ELP and Milli-Q water at 60 °C yielded NEs of 53.3 ± 0.2 nm hydrodynamic diameter and 0.13 ± 0.02 polydispersity, according to dynamic light scattering (DLS, Table S1 and Fig. S6). In order to make an independent verification of the reaction between NPC-Chol and Biotin-PEG_3000_-Lysine, absorption spectra of the obtained NEs mixtures were recorded in order to identify the presence of released *p*-nitrophenol. The reaction mixture exhibited a peak around 400 nm, characteristic of *p*-nitrophenol absorption,^34^ whereas the control experiment without the linker showed negligible contribution of *p*-nitrophenol absorption. Thus, we confirmed that the reaction took place, based on the release of *p*-nitrophenol.

Then, to quench the non-reacted NPC-Chol, we incubated the NEs mixture with an excess of *n*-butylamine, which led to further increase in the absorbance of *p*-nitrophenol. The reaction was incubated until no significant increase in absorbance was observed (Fig. 2B). Assuming that after incubation with *n*-butylamine, 100% of NPC-Chol reacted with the amines, liberating all *p*-nitrophenol, we could estimate that 17.1% of the total NPC-Chol reacted with Biotin-PEG_3000_-Lysine. This estimation is in line with the NMR data above, confirming the formation of the linker-cholesterol conjugate. Then, the mixture was dialyzed to remove non-reacted linker, non-reacted butylamine, and liberated *p*-nitrophenol. DLS of the dialyzed product suggested the presence of nano-droplets of 64 ± 4 nm diameter with a polydispersity index of 0.2 ± 0.03 (Table S1). According to zeta potential measurements, NEs with and without biotin linker were neutral (Table S1), as particles with zeta potential values of ± 10 mV are considered to have neutral surfaces.^35^ This result is expected for non-functionalized and biotinylated NEs, given that their surface exposed components, such as Kolliphor ELP and the PEGylated biotin linker, are neutral. Altogether, we developed a one-pot green chemistry approach for NEs biotinylation using GRAS components, thereby reducing the risk of ligand detachment from the NEs surface.

**Fig. 3:**
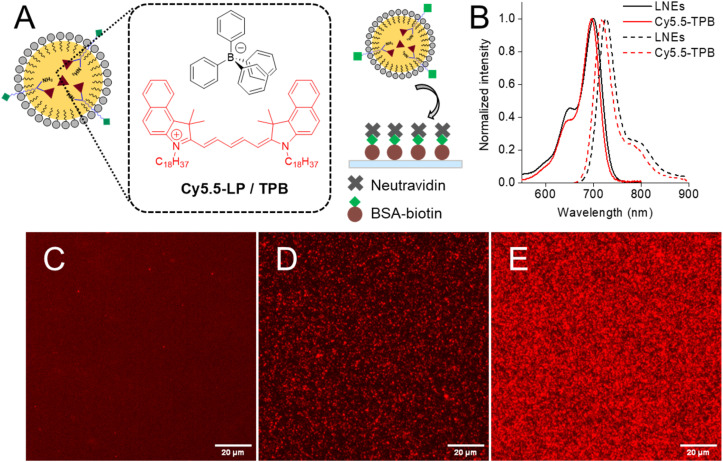
Validation of biotinylated nanoemulsions. (A) Schematic representation of Cy5.5-LP/TPB loaded NEs and their immobilization on a glass surface coated with BSA-biotin and neutravidin. (B) Absorption (solid line) and emission (dashed line) spectra of NEs loaded with Cy5.5-LP/TPB in water, and Cy5.5-LP/TPB in Labrafac. (C–E) Fluorescence microscopy of neutravidin-coated glass surface incubated with bare NEs (30 000-fold dilution) (C) or biotin-decorated NEs (100 000- (D) or 30 000-fold (E) dilution). Scale bar = 20 μm.

As a model of hydrophobic cargo and a fluorescent tracer of NEs, we added to the oil core lipophilic fluorescent dye – cyanine 5.5 derivative with its bulky counterion tetraphenylborate (Cy5.5-LP/TPB, Fig. 3A).^8^ The TPB counterion has been shown to be essential to ensure efficient encapsulation of the dye within NEs with minimal dye leakage and aggregation-caused quenching.^8,11^ The absorption and emission spectra of these NEs exhibited single bands with maxima at 700 and 727 nm, which were close to those for Cy5.5-LP in labrafac oil (Fig. 3B). Absorption spectra non-functionalized and biotin NEs (Fig. S7) allowed us to estimate that the dye encapsulation efficiencies were 94% and 79%, respectively, relative to theoretical 2 wt% dye loading. The high encapsulation efficiency in case of non-functionalized NEs is expected because highly lipophilic Cy5.5-LP/TPB is expected to remain within the oil core during formulation. However, the lower loading efficiency observed for biotin-NEs could be related to partial loss of the dye under the biotinylation reaction conditions. To evaluate the stability of the cargo encapsulation inside NEs, we studied the effect of dialysis on the absorbance of the encapsulated Cy5.5-LP/TPB dye. However, only minor decrease of the absorbance (<20%) was observed for both non-functionalized and biotin NEs after the dialysis (Fig. S7). The latter indicates that the dye is stably encapsulated and can resist to dialysis, clearly because of its high lipophilicity. Nevertheless, the small part of the dye was probably gone together with the excess of the surfactant (Kolliphor ELP), thus explaining the observed small decrease in the absorbance. To further understand the stability of the formulations of NEs in biological media and potential release of the cargo (dye), we performed fluorescence correlation spectroscopy (FCS),^36^ the method commonly used for characterization of NPs in complex media.^37–40^ Here, we incubated our NEs in standard phosphate buffer saline (DPBS) and in buffer with fetal bovine serum (10 vol%) at RT and 37 °C. As NEs of 50 nm diameter should contain ∼600 dyes per particle, we expect that the leakage of dyes into serum should generate manifold increase in the number of emissive species.^37^ However, our data showed that incubation of non-functionalized and biotin NEs resulted in negligible increase in the number of emissive species (Fig. S8). These results suggest that NEs are stable in biological media with almost negligible release of the dye cargo, in line with previous studies of this dye inside NEs.^8^ Moreover, biotin NEs (without dye) were stored at 4 °C for one year, and the DLS measurements (Table S1 and Fig. S6) suggested minimal changes in their hydrodynamic diameter throughout this period, indicating good storage stability.

### Validation of surface biotin activity *via* neutravidin binding assay

To assess the presence of active biotin units on the surface of NEs, we formulated biotinylated-NEs loaded with a hydrophobic fluorescent dye Cy5.5-LP/TPB at 2 wt% in the oil phase (Fig. 3A). According to DLS, the obtained Cy5.5-loaded biotinylated NEs were 53 ± 2 nm in diameter, with a polydispersity index of 0.16 ± 0.01 (Table S1). Using this Cy5.5-labeling, we characterized the biotinylated-NEs immobilized on a glass surface at the single-particle level by fluorescence microscopy (Fig. 3B). As a conjugation biomolecule for biotin, we used neutravidin, an avidin analogue presenting higher selectivity and lower non-specific binding.^41^ Hence, neutravidin is frequently used for immobilization of biotinylated molecules and nanoparticles on the surface.^24,31,42,43^ To test interaction of neutravidin with biotin on NEs surface, we coated a glass surface with BSA-biotin, followed by a layer of neutravidin based on established protocols (Fig. 3A).^42,43^ With this surface at hand, we compared targeting specificity of biotinylated-NEs with control NEs without grafted biotin. Biotinylated-NEs were clearly visible on the glass surface by fluorescence microscopy in the form of dots (Fig. 3D and E). The density of dots and the overall intensity of the signal in the images increased with higher NEs concentration (Fig. 3D and E). In the case of control non-modified NEs, fluorescent dots were absent already for the highest used NEs concentration (Fig. 3C). These results clearly show that the grafted biotin remains accessible and functionally active, as evidenced by its specific interaction with neutravidin, while non-specific interactions with the NEs with the surface are negligible.

### Functionalization of NEs with trastuzumab

**Fig. 4:**
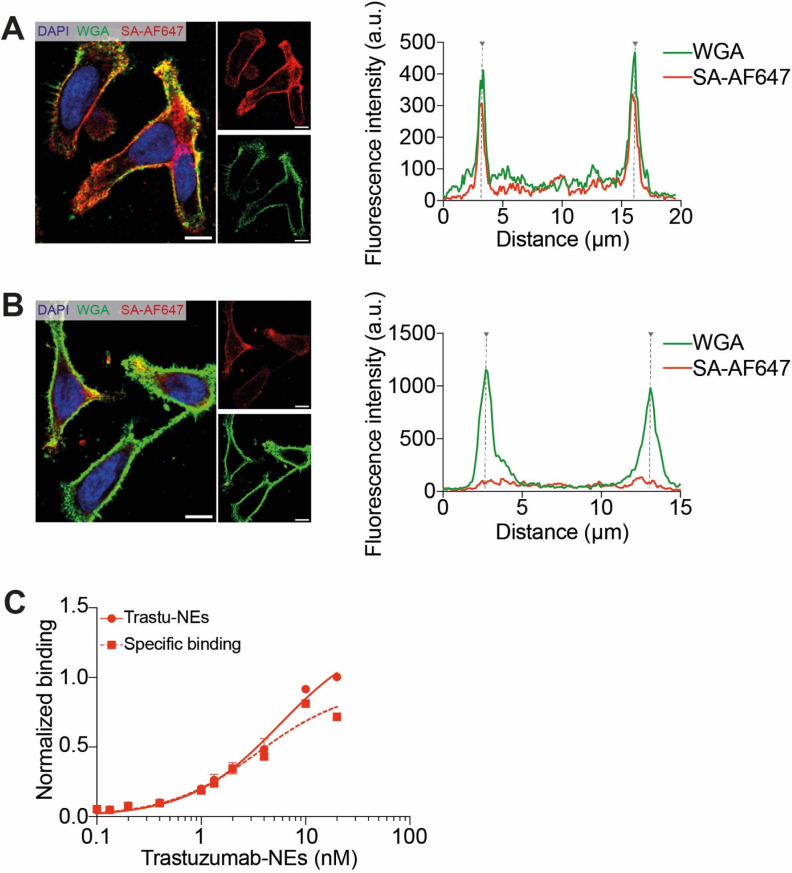
Validation of biotinylated trastuzumab on HER2-amplified HCC1954 cells. (A and B) Quantification of the trastuzumab-biotin membrane staining by following Streptavidin-AF647 (SA-AF647) fluorescence without (A) or with (B) pre-blocking with neutravidin, using fluorescence confocal microscopy. Cell membranes were labeled with Wheat Germ Agglutinin-AF488 (green). Scale bar is 10 μm. Cell edges are marked by dot lines on the fluorescence profil plots. (C) Flow cytometry study determining the apparent binding affinity of the trastuzumab-functionalized nanoemulsions on HER2-amplified HCC-1954 cells. Error bars are standard deviation of the mean (*n* = 2).

Prompted by these results, we next generated NEs decorated with therapeutic antibodies to enable selective targeting of tumor cells expressing cognate antigens. For this purpose, trastuzumab – a clinically approved anti-HER2 antibody^34^ – was conjugated to biotin *via* its amino groups at physiological pH using a biotin-NHS ester,^34^ and unreacted reagent was removed by ultrafiltration. Efficient biotinylation was confirmed by strong membrane staining in a cell binding assay on HER2-amplified HCC1954, using Streptavidin-AF647 (SA-AF647) to detect accessible biotin sites (Fig. 4A). The specificity of the biotin–streptavidin interaction was validated by pre-blocking with neutravidin, which abolished membrane-localized fluorescence (Fig. 4B). Next, biotinylated trastuzumab was coupled to biotinylated NEs *via* a neutravidin bridge. This interaction is rapid, highly specific, and extremely stable (*K*_d_ ≈ 10^−15^ mol L^−1^).^30,31^ Unbound antibodies were then removed by size exclusion chromatography using Sephacryl S300 HR, which is appropriate for separating relatively large macromolecules or globular proteins in the range of 10 to 150 kDa. UV-visible absorbance spectra of the eluted fractions showed that trastuzumab-decorated NEs were recovered in early fractions (f3–f5), with coincident absorbance at 280 nm (protein) and 700 nm (Cy5.5-LP) (Fig. S9). In contrast, free trastuzumab eluted in later fractions (f6–f8), showing absorbance only at 280 nm. This result suggests that the first fraction presenting both 280 and 700 nm absorbance (f3–f5) correspond to both NEs and conjugated trastuzumab, while the following fractions correspond to proteins (free antibodies and possibly neutravidin). The first fractions were combined and studied again by the same chromatography method. This time only a one band corresponding to the first fractions was observed, presenting absorbance in both 280 and 700 nm (Fig. S10). Thus, size exclusion method allows separation of the of NEs from non-conjugated protein molecules. Importantly, antibody functionalization had negligible impact on NE hydrodynamic diameter (56 ± 2 nm) with preserved good polydispersity of 0.12 ± 0.03 (Table S1). Zeta potential measurements suggested that the zeta potential was only slightly negative (−6.2 ± 0.8 mV, Table S1). While trastuzumab has an isoelectric point (pI) ranging from 8.0 to 8.6,^44^ neutravidin has an isoelectric point of 6.3 (according to provider Thermo Scientific). Therefore, the resulting zeta potential depends on the stoichiometry of the conjugate. However, assuming the pI of trastuzumab-neutravidin conjugate should exhibit intermediate value of those for the free species, the conjugate would be neutral at pH ∼7, thus explaining minimal changes in the zeta potential after modification of NEs with neutravidin–trastuzumab conjugate. To confirm that trastuzumab conjugation did not impair antigen recognition, we determined the apparent affinity constant (*K*_d_) of trastuzumab-decorated NEs by flow cytometry, using biotinylated NEs without antibody as a negative control. Fitting the binding data to a one-site Langmuir isotherm yielded an apparent *K*_d_ of 3.5 ± 1.8 nmol L^−1^ (Fig. 4C), consistent with previously reported values for trastuzumab in cell-based assays.^45,46^

### Trastuzumab-functionalized NEs target HER2-amplified tumor cells *in vitro* with high selectivity

**Fig. 5:**
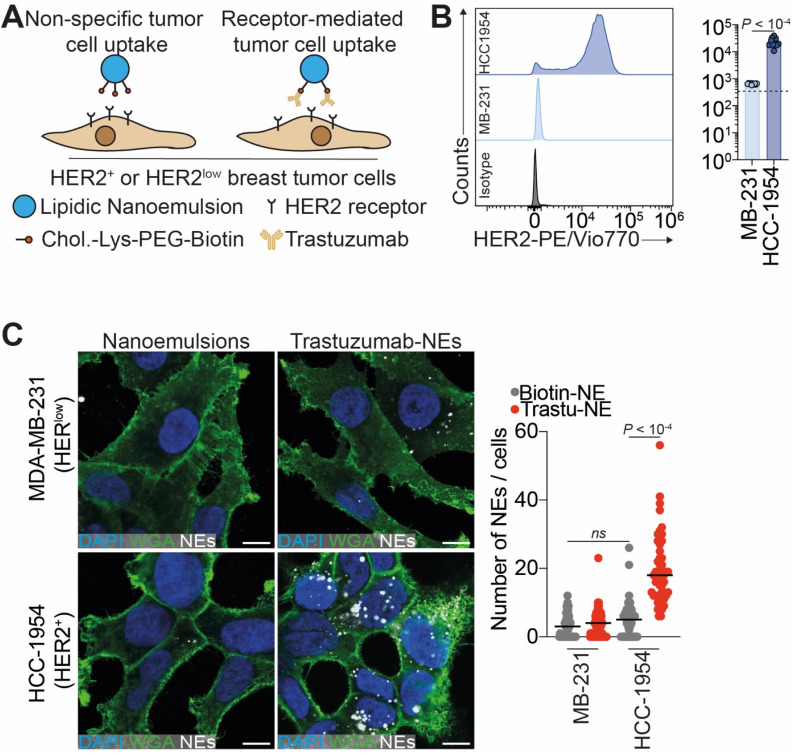
Trastuzumab-functionalized nanoemulsions target breast cancer cells in an antigen–antibody manner. (A) Biotinylated-NEs or trastuzumab-functionalized NEs interactions, assessed using HER2-amplified HCC-1954 cells or HER2^low^ MDA-MB-231 cells. (B) Flow cytometry analysis of HCC-1954 and MDA-MB-231 HER2 status. (C) Imaging (left) and quantification (right) of the nanoemulsion uptake in HER2^low^ (MDA-MB-231) and HER2-amplified (HCC-1954) cells using confocal fluorescence microscopy. Cells incubated for 3 h with 1.1 nM of Cy5.5-loaded (red) biotinylated NEs or trastuzumab-targeted NEs. Cell membranes were labeled with Wheat Germ Agglutinin-AF488 (green). Scale bar is 10 μm. In the data quantification (right), number of MDA-MB-231 cells per condition: 47 for 112 for trastuzumab-NEs; number of HCC1954 cells per condition: 61 for NEs and 51 for trastuzumab-NEs.

**Fig. 6:**
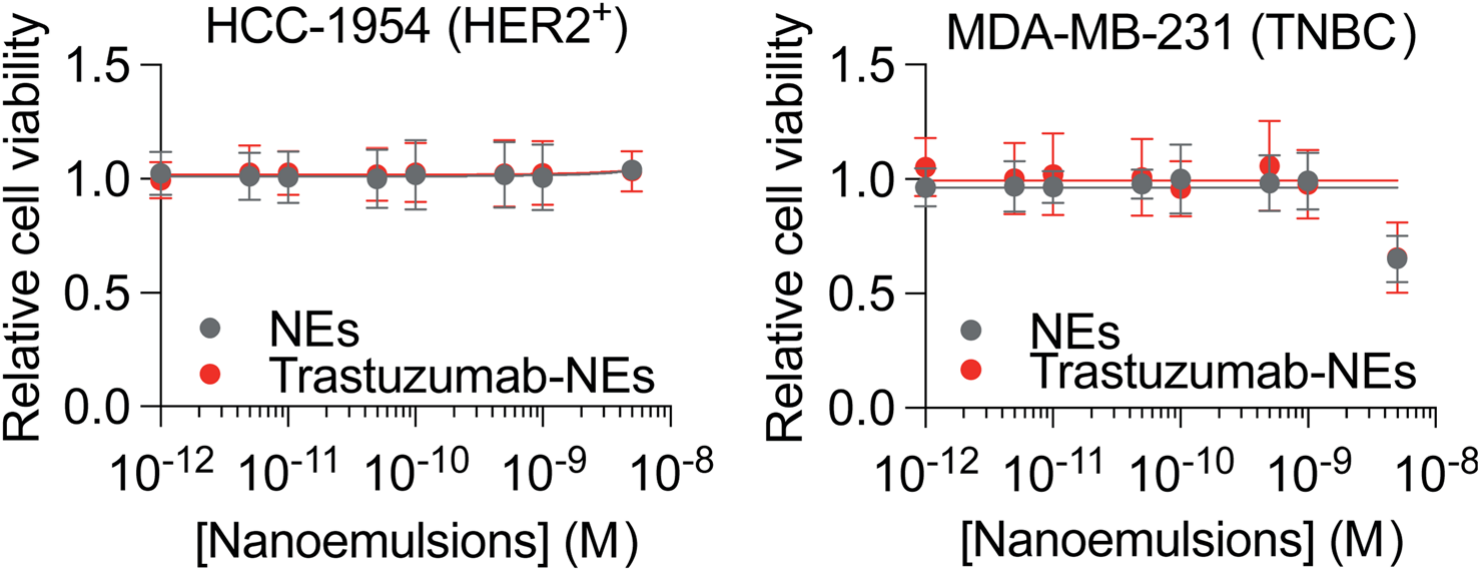
Nanoemulsions cytotoxicity, measured after a 72 h treatment with NEs increasing concentrations of HER2-amplified HCC-1954 (left) and HER2^low^ MDA-MB-231 (right) cells. Cytotoxicity was assessed using a crystal violet staining. Error bars are standard déviation of the mean (*n* = 3 for MDA-MB-231 and *n* = 5 for HCC1954).

Building on these results, we next evaluated whether trastuzumab-functionalized NEs can target specifically tumor cells with high expression of HER2 receptor (HCC-1954) (Fig. 5A and B) using confocal fluorescence imaging, of NEs loaded with Cy5.5-LP (2 wt% *vs.* oil). Trastuzumab-functionalized NEs showed markedly increased internalization in HER2-amplified HCC-1954 cells compared to passive uptake by biotinylated NEs alone (Fig. 5C). To confirm HER2 dependency, we assessed internalization in HER2^low^ MDA-MB-231 cells, where no detectable differences were observed between targeted and untargeted NEs (Fig. 5C). To further validate the targeting selectivity, we extended the analysis to an additional HER2-positive cell line, SKBR3, which also showed preferential uptake of trastuzumab-functionalized NEs over control formulations (Fig. S11). To evaluate the biocompatibility of the formulation approach, we assessed whether NEs uptake induced cytotoxic effects. Treating both HER2-amplified and HER2^low^ cell lines with either trastuzumab-targeted or untargeted NEs had no effect on cell viability (Fig. 6). Altogether, these results demonstrate the enhanced targeting selectivity of trastuzumab-functionalized NEs toward HER2-expressing tumor cells. This strategy provides a versatile and biocompatible platform for antibody-based surface functionalization of NEs to improve tumor-targeting efficiency.

## Conclusions

Functionalization of lipid NEs with biomolecules for selective targeting remains a challenge. Here, we developed an original plug-and-play strategy to graft an antibody (trastuzumab) at the surface of NEs, using GRAS components. For this purpose, we synthesized a reactive derivative of cholesterol, cholesteryl (4-nitrophenol) carbonate (NPC-Chol), which can be readily encapsulated in the oil core of NEs and react with amines. We also designed a Biotin-PEG_3000_-Lysine linker with two amino groups of lysine at one end for reaction with NPC-Chol to form the amphiphilic carbamate Biotin-PEG_3000_-Chol. The cholesterol moiety was expected to ensure its firm anchorage in NEs. On the other end of the linker, the long hydrophilic PEG_3000_ chain is expected to expose the biotin moiety at the surface of NEs for further antibody grafting using a biotin–neutravidin coupling. The reaction between the Biotin-PEG_3000_-Lysine linker and NPC-Chol was confirmed by ^1^H-NMR and absorption spectroscopy. Using the one-pot approach, the obtained linker cholesterol conjugate, along with the corresponding oil and surfactant, was formulated into 50-nm NEs, which were expected to bear biotin ligands. The successful biotinylation was confirmed by fluorescence microscopy: biotinylated-NEs loaded with a near-infrared dye were successfully targeted to neutravidin-coated glass surfaces in contrast to non-modified NEs. The obtained fluorescent biotinylated-NEs were grafted with biotinylated anti-HER2 (trastuzumab) monoclonal antibodies *via* a neutravidin bridge. The described approach presents several unique features (Table S2). Compared to the previously proposed method based on amphiphilic polymers^24,25^ the present approach uses GRAS and biodegradable components, addressing safety concerns. On the other hand, compared to methods based on PEGylated lipids,^21,47^ the use of cholesterol increases the strength of anchoring with a lower chance of exchange in biological medium, due to the higher hydrophobicity of cholesterol. Additionally, the use of a long spacer ensures proper display of the biotin moiety away from the NE surface, unlike methods based on fatty acids.^22,48^

The targeting selectivity of the trastuzumab-decorated NEs was evaluated *in vitro* by confocal microscopy on relevant breast cancer models HCC-1954 and SKBR3 (HER2-amplified) and MDA-MB-231 (HER2^low^). Targeted NEs presented higher cellular internalization compared to control NEs in corresponding positive cell lines. In contrast, in control cell lines with low HER2 expression, no internalization was observed for both targeted and non-targeted formulations. These results showed a receptor-dependent targeting for our NEs functionalized with antibodies.

Altogether, our study showed that combining the high encapsulation capacity of the NEs with the trastuzumab surface decoration allowed receptor-selective targeting of HER2-amplified breast cancer cells. This easily tunable functionalized system provides a platform for the conjugation of different monoclonal antibodies and the encapsulation of a large variety of contrast agents and drugs; thus, it can be used for imaging, therapeutic and targeted drug delivery applications. Although *in vivo* validation is pending, nanoemulsions with similar composition and properties have demonstrated favorable pharmacokinetics, including extended circulation and organ-specific accumulation *via* the EPR effect.^8,49,50^ Given their biocompatibility, scalability, and proven versatility in drug delivery, our system holds strong potential for clinical translation. However, further improvements are still required. Indeed, the streptavidin analogue neutravidin used to bridge antibodies to NEs can be immunogenic^51^ and could eventually be replaced by direct biorthogonal conjugation methods such as click chemistry.^25,52,53^ In this case, the same green chemistry could be applied, where the biotin unit is replaced with corresponding reactive group. This work is currently in progress. In addition, the NEs used in this study are lipidic nanoparticles and thus should, according to our meta-analysis results, be targeted with fragments rather than full antibodies for significantly higher accumulation.^14^ These improvements will be further complemented with *in vivo* experiments of mice bearing tumor models in order to evaluate the full potential of functionalized NEs in biomedical applications.

## Conflicts of interest

The authors have no conflict of interest to declare.

## Supplementary Material

NA-007-D5NA00606F-s001

## Data Availability

The data supporting this article have been included as part of the supplementary information (SI). Supplementary information: it contains NMR and mass spectra, additional data on DLS, zeta potential, optical spectroscopy, FCS, chromatography analysis and cell imaging and comparative analysis of developed functionalization approach with respect to previously reported ones. See DOI: https://doi.org/10.1039/d5na00606f.
