# Supplementary material for: Functionalization of lipid nanoemulsions with humanized antibodies using plug-and-play cholesterol anchor for targeting cancer cells
Source: Nanoscale Adv. 2025 Sep 23;7(22):7226–38. doi: 10.1039/d5na00606f (PMC12489389; doi:10.1039/d5na00606f)
Supplement: NA-007-D5NA00606F-s001 [file NA-007-D5NA00606F-s001.pdf]

Supplementary Information (SI) for Nanoscale Advances.
This journal is © The Royal Society of Chemistry 2025

# Supporting information

# Functionalization of lipid nanoemulsions with humanized antibodies using plug-and-play cholesterol anchor for targeting cancer cells

**Valeria Jose Boide-Trujillo[1,#], Vincent Mittelheisser[2,#], Fei Liu[1,3,#], Olivier Lefebvre[2], Bohdan Andreiuk[1], Nicolas Anton[3], Jacky G. Goetz[2,*], and Andrey S. Klymchenko[1,*]**

[1]Laboratoire de Bioimagerie et Pathologies, CNRS UMR_7021, ITI SysChem-Chimie des Systèmes Complexes, Université de Strasbourg; Illkirch (France)

[2]Tumor Biomechanics Lab, INSERM UMR_S1109, Fédération de Médecine Translationnelle de Strasbourg, Université de Strasbourg, Equipe labélisée Ligue Nationale Contre le Cancer ; Strasbourg (France).

[3]Regenerative Nanomedicine (RNM), INSERM UMR_1260, Fédération de Médecine Translationnelle de Strasbourg, Université de Strasbourg; Strasbourg (France)

[#]**Equal contribution:**
VJBT, VM and FL have contributed equally to this work.

*__Correspondence and co-last authors:__
Andrey S. Klymchenko, andrey.klymchenko@unistra.fr
CNRS UMR_7021, Faculté de Pharmacie, Université de Strasbourg, Institut Thématique Interdisciplinaire SysChem, Illkirch F-67400, France.

Jacky G. Goetz, jacky.goetz@inserm.fr
INSERM UMR_S1109, Tumor Biomechanics Lab, Centre de Recherche en Biomédecine de Strasbourg (CRBS), Fédération de Médecine Translationnelle de Strasbourg (FMTS), 67000 Strasbourg, France. Web: www.goetzlab.fr

## Supplementary Figures

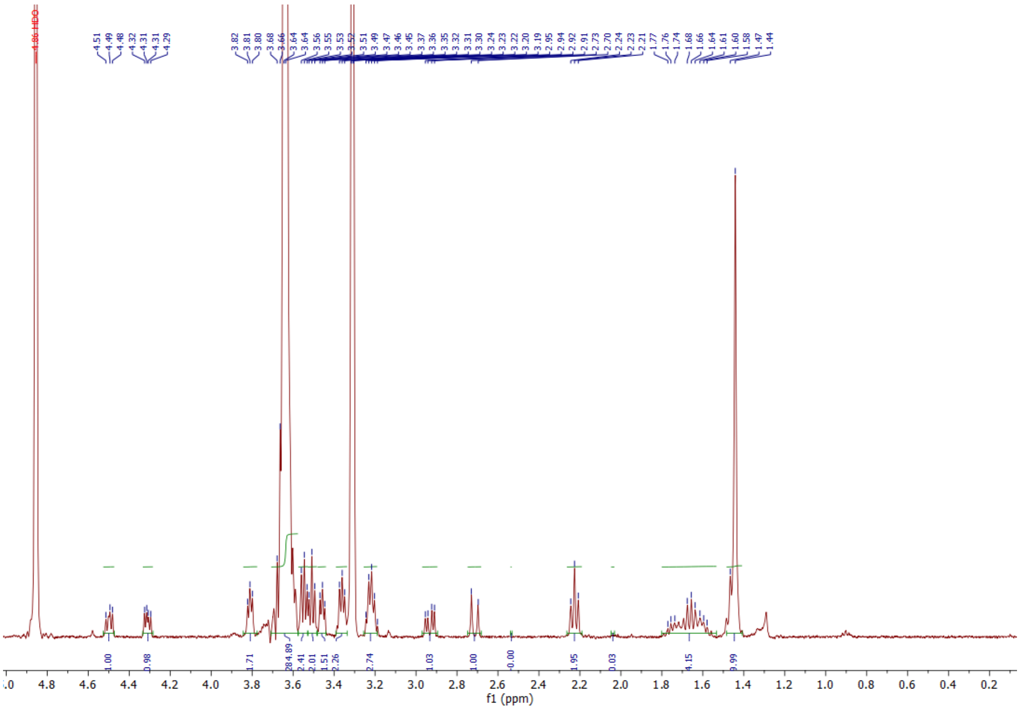


**Figure S1.** $^{1}$H-NMR of compound **1** in $CD_3OD$.

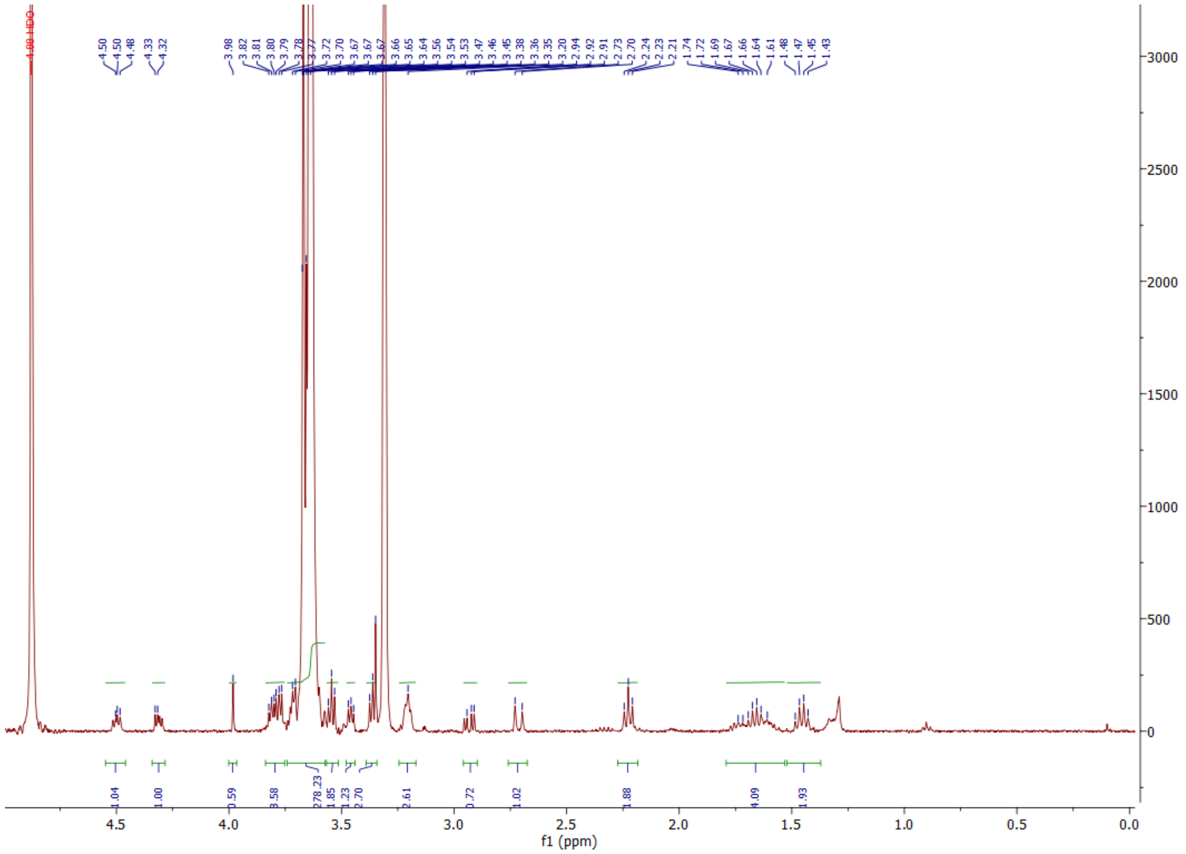

**Figure S2.** $^{1}$H-NMR of compound **2** in $CD_3OD$.

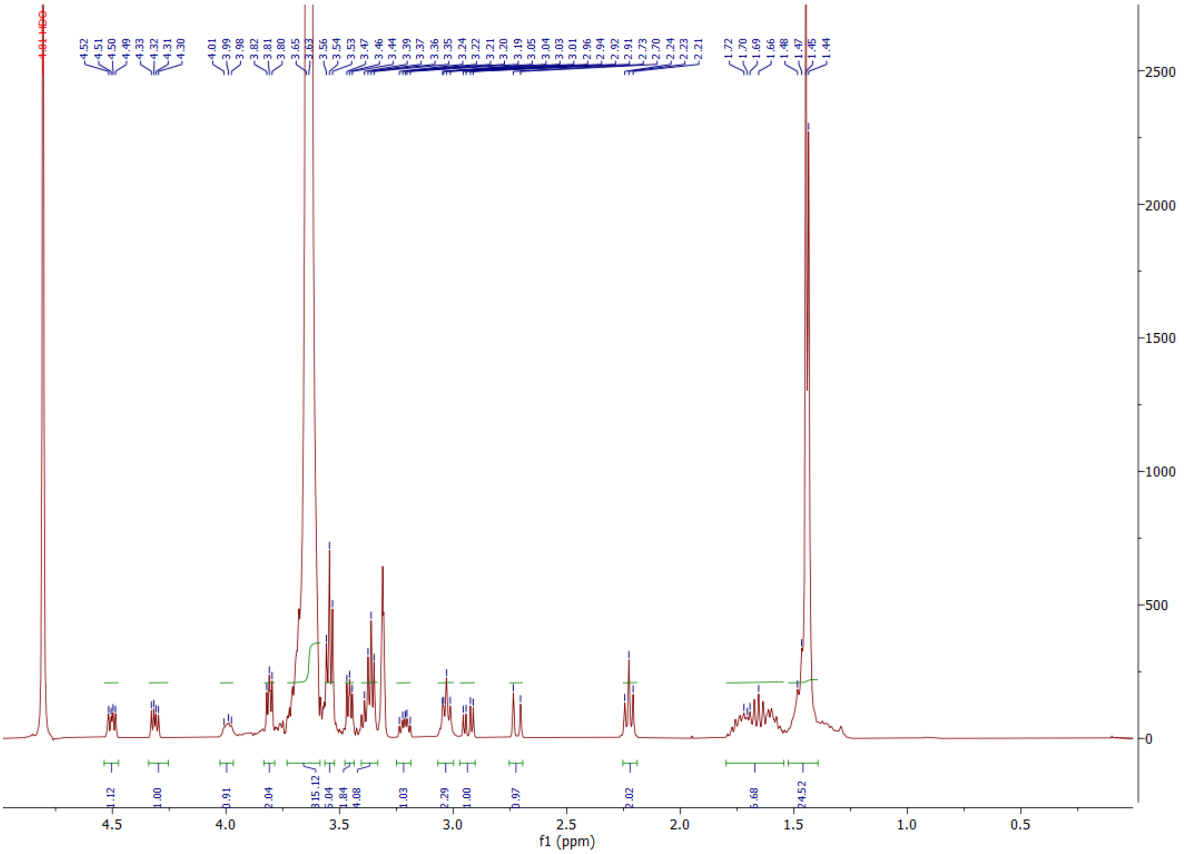

**Figure S3.** $^{1}$H-NMR of compound **3** in $CD_3OD$.

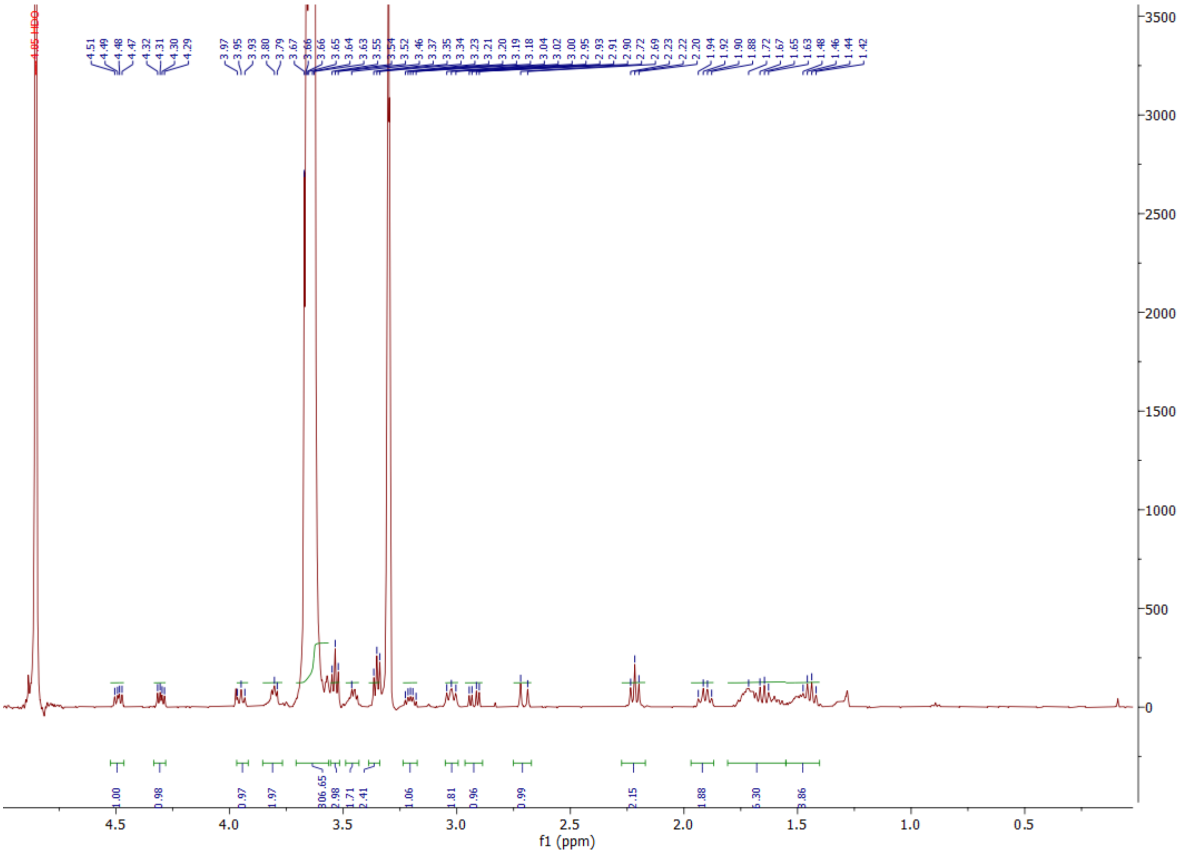

**Figure S4.** $^1$H-NMR of compound **4** in $CD_3OD$.

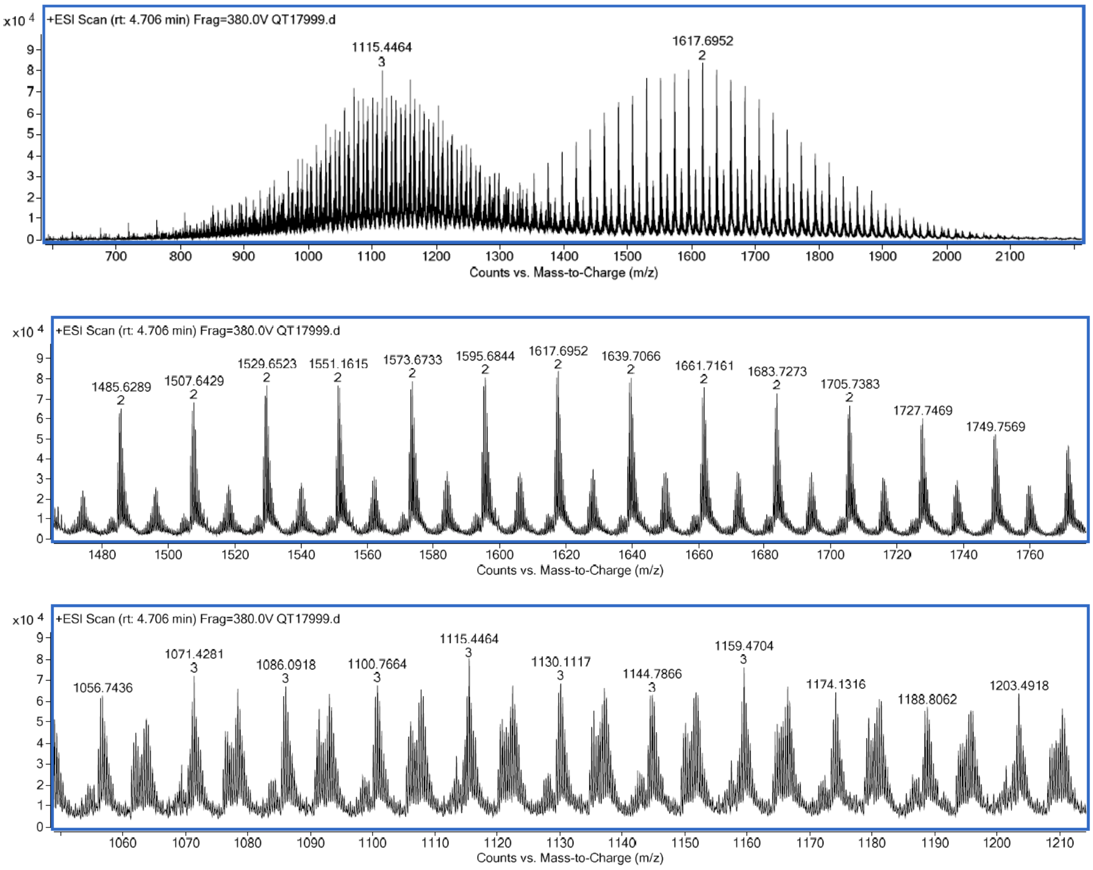


**Figure S5.** Mass spectra of compound **4** in $CD_3OD$.

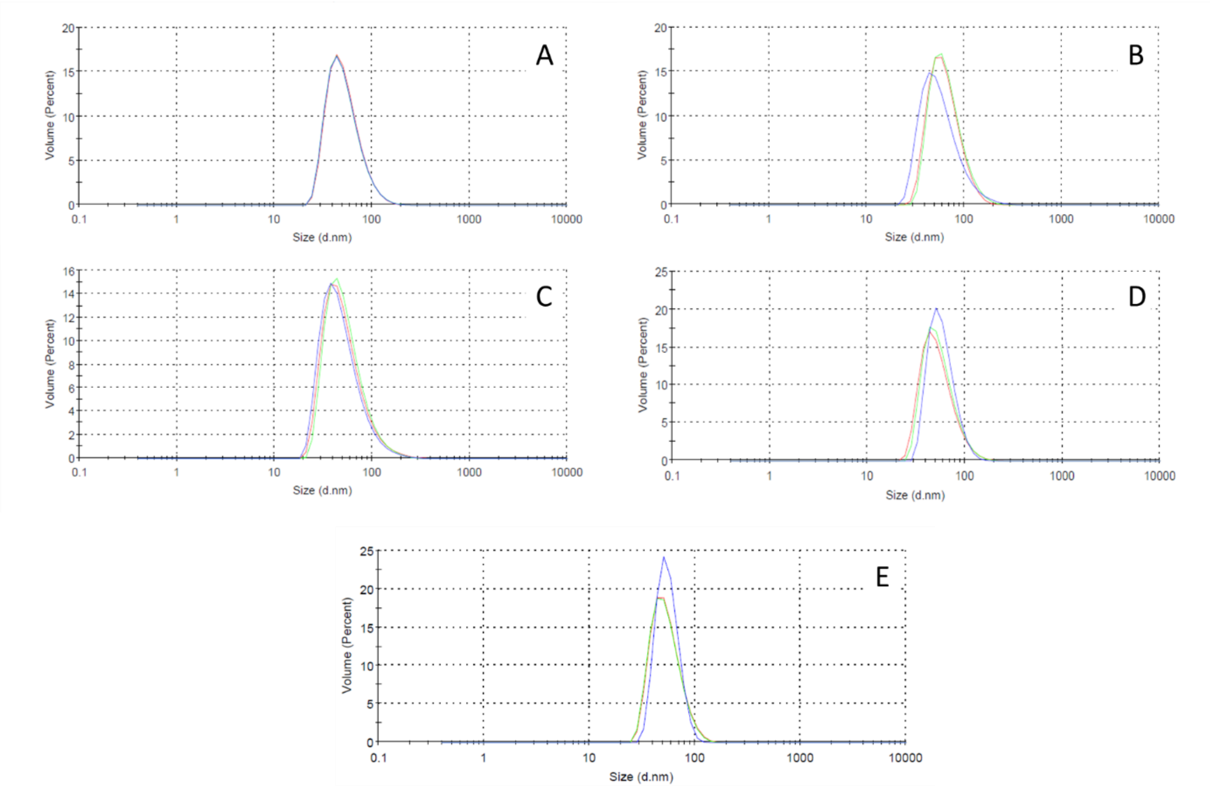


**Figure S6.** Size distribution by volume obtained by dynamic light scattering for: (A) non-functionalized NEs, (B) biotin-NEs (no dye), (C) biotin-NEs (with dye), (D) antibody-NEs, and (E) biotin-NEs (no dye) after one year of storage at +4 °C. Data correspond to those in Table S1.

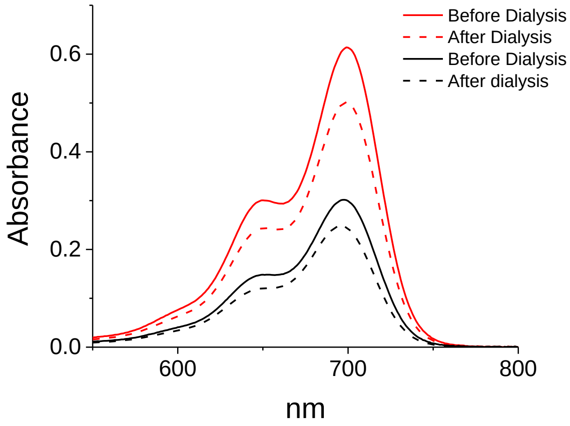


**Figure S7.** Absorption spectra of non-functionalized NEs (red) and biotin-NEs (black) before and after dialysis.

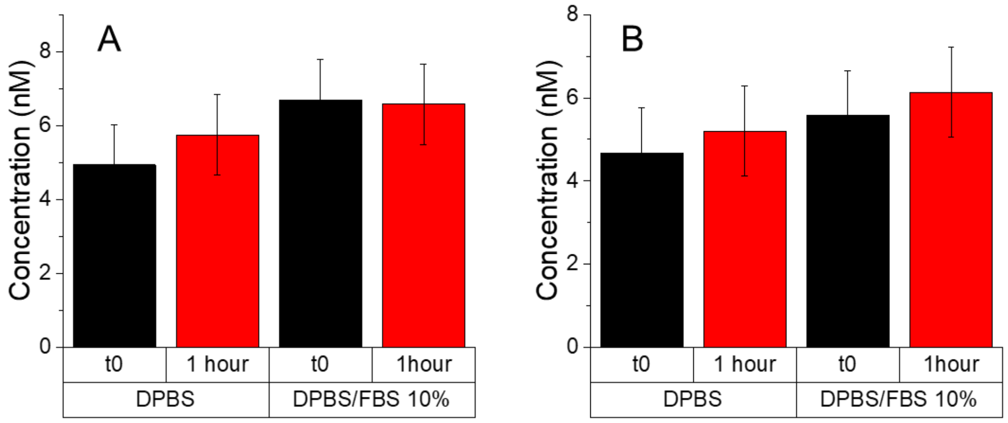


**Figure S8.** FCS studies of fluorophore release (leakage) in DPBS and FBS 10%/DPBS, based on evaluation of absolute concentration of Cy5.5-TPB loaded biotin-NEs (A) and non-functionalized NEs (B) after 1 hour of incubation at 37 °C or directly diluted into the buffer without incubation. Error bars are standard deviation of the mean, based on 20-35 FCS runs for the same sample. For calculation of concentration, Alexa-647 (50 mM concentration) was used as a reference.

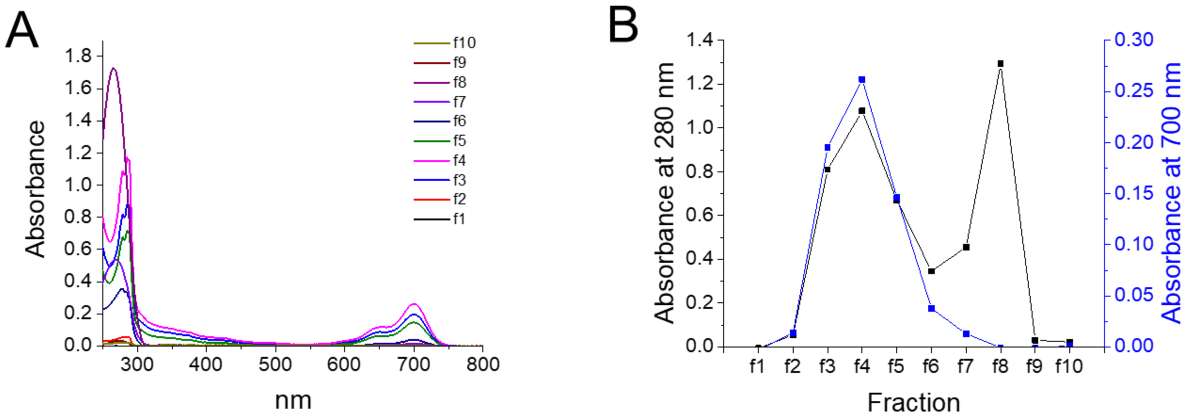


**Figure S9.** (A) Absorption spectra of different fractions of non-purified mixture containing NEs, neutravidin and biotinylated mAbs. (B) Absorbance value for different fractions recorded at 280 and 700 nm.

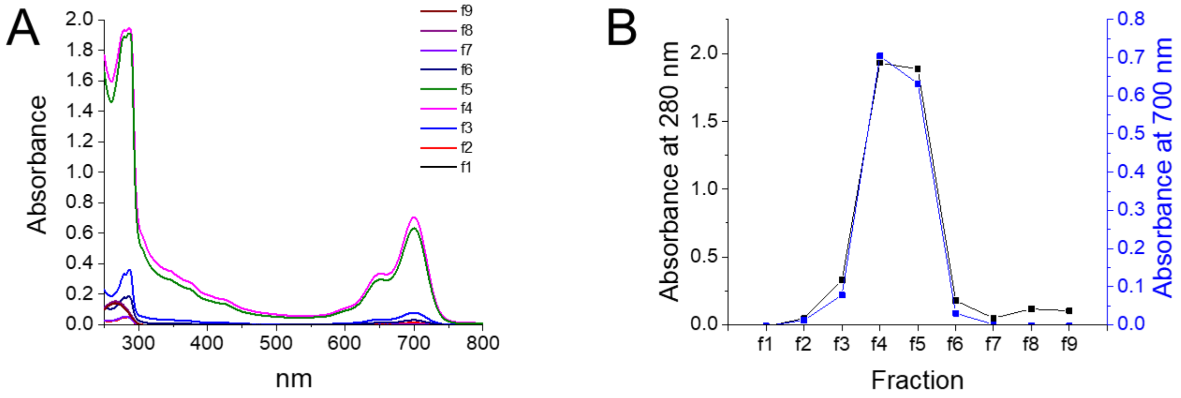


**Figure S10.** (A) Absorption spectra of different fractions of NEs conjugated with mAbs after purification. (B) Absorbance value for diff1erent fractions recorded at 280 and 700 nm.

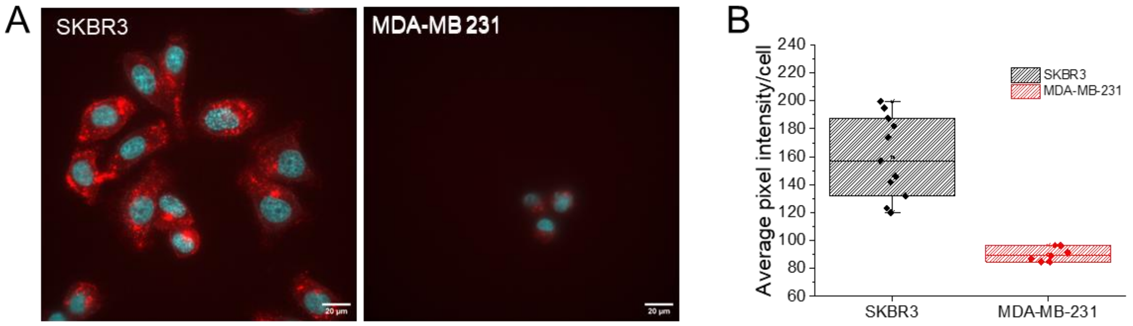


**Figure S11.** (A) Epifluorescence microscopy images from SKBR3 and MDA-MB 231 cells after 1h incubation on ice with Antibody-NEs (0.2 nM) containing 2% of Cy5.5-TPB. (B) Quantitative image analysis.

**Supplementary Tables**

**Table S1.** Size of NEs measured by DLS.

| NEs type | Diameter (nm) | Polydispersity index (PdI) | Zeta Potential (mV) |
|---|---|---|---|
| Non-functionalized (6 wt. % NPC-Chol and 2 wt. % Cy5.5-LP/TPB) | 53.3 ± 0.2 | 0.13 ± 0.02 | -2.5 ± 0.3 |
| Biotinylated (no dye) | 64 ± 4 | 0.20 ± 0.03 | -2.6 ± 0.1 |
| Biotinylated (no dye) after 1 year at 4 °C | 55 ± 1 | 0.14 ± 0.01 | - |
| Biotinylated (2 wt. % Cy5.5-TPB) | 53 ± 2 | 0.16 ± 0.01 | -2 ± 1 |
| Antibody (2 wt. % Cy5.5-TPB) | 56 ± 2 | 0.12 ± 0.03 | -6.2 ± 0.8 |

Note: all diameter and polydispersity index values shown are the mean values±standard deviation based on three measurements of the same sample. All zeta potential potential data are the mean values±standard deviation based on five measurements of the same sample.

**Table S2.** Comparison of developed approach with previously reported functionalization strategies.

| Feature | Present work | Previous reports | Ref. |
|---|---|---|---|
| **Materials used** | GRAS and biodegradable components | Non-biodegradable amphiphilic polymers | [1, 2] |
| **Safety** | Improved (safer materials) | Potential safety concerns with polymers | |
| **Anchoring stability** | High (cholesterol is more hydrophobic) | PEGylated phospholipids are prone to exchange | [3, 4] |
| **Active moiety exposure** | Far from NEs surface thanks to PEG spacer | Close to NEs surface | [5, 6] |

## Referenes


1. S. Bou, X. Wang, N. Anton, R. Bouchaala, A. S. Klymchenko and M. Collot, Lipid-core/polymer-shell hybrid nanoparticles: synthesis and characterization by fluorescence labeling and electrophoresis, *Soft Matter*, 2020, **16**, 4173-4181.
2. E. Belcastro, A. U. Rehman, L. Remila, S.-H. Park, D. S. Gong, N. Anton, C. Auger, O. Lefebvre, J. G. Goetz, M. Collot, A. S. Klymchenko, T. F. Vandamme and V. B. Schini-Kerth, Fluorescent nanocarriers targeting VCAM-1 for early detection of senescent endothelial cells, *Nanomedicine: Nanotechnology, Biology and Medicine*, 2021, 102379-102379.
3. M. Goutayer, S. Dufort, V. Josserand, A. Royère, E. Heinrich, F. Vinet, J. Bibette, J. L. Coll and I. Texier, Tumor targeting of functionalized lipid nanoparticles: assessment by in vivo fluorescence imaging, *Eur J Pharm Biopharm*, 2010, **75**, 137-147.
4. S. Bonnet, G. Prévot, S. Mornet, M.-J. Jacobin-Valat, Y. Mousli, A. Hemadou, M. Duttine, A. Trotier, S. Sanchez, M. Duonor-Cérutti, S. Crauste-Manciet and G. Clofent-Sanchez, A Nano-Emulsion Platform Functionalized with a Fully Human scFv-Fc Antibody for Atheroma Targeting: Towards a Theranostic Approach to Atherosclerosis, *International Journal of Molecular Sciences*, 2021, **22**, 5188.
5. M. F. Attia, M. I. Swasy, R. Akasov, F. Alexis and D. C. Whitehead, Strategies for High Grafting Efficiency of Functional Ligands to Lipid Nanoemulsions for RGD-Mediated Targeting of Tumor Cells In Vitro, *ACS Applied Bio Materials*, 2020, **3**, 5067-5079.
6. S. A. Navarro-Marchal, C. Griñán-Lisón, J.-M. Entrena, G. Ruiz-Alcalá, M. Tristán-Manzano, F. Martin, I. Pérez-Victoria, J. M. Peula-García and J. A. Marchal, Anti-CD44-Conjugated Olive Oil Liquid Nanocapsules for Targeting Pancreatic Cancer Stem Cells, *Biomacromolecules*, 2021, **22**, 1374-1388.
